# The prevalence of current smokers and alcohol drinkers among cancer survivors and subjects with no history of cancer among participants in a community‐based cardiometabolic screening program in Miyagi prefecture, Japan: a comparison with nationally representative surveys in other countries

**DOI:** 10.1002/cam4.4364

**Published:** 2021-12-01

**Authors:** Yuka Nishimoto, Yoshitaka Tsubono, Mana Kogure, Tomohiro Nakamura, Fumi Itabashi, Naho Tsuchiya, Naoki Nakaya, Kozo Tanno, Junichi Sugawara, Shinichi Kuriyama, Shigeo Kure, Ichiro Tsuji, Atsushi Hozawa

**Affiliations:** ^1^ Division of Personalized Prevention and Epidemiology Tohoku Medical Megabank Organization Tohoku University Sendai Japan; ^2^ Epidemiology and Prevention Group Center for Public Health Sciences National Cancer Center Tokyo Japan; ^3^ Iwate Tohoku Medical Megabank Organization Disaster Reconstruction Center Iwate Medical University Yahaba Japan; ^4^ School of Medicine Iwate Medical University Morioka Japan; ^5^ Department of Pharmaceutical Sciences Tohoku University Hospital Sendai Japan; ^6^ Department of Molecular Epidemiology Graduate School of Medicine Tohoku University Sendai Japan; ^7^ Department of Disaster Public Health, International Research Institute of Disaster Science Tohoku University Sendai Japan; ^8^ Department of Pediatrics Tohoku University School of Medicine Sendai Japan; ^9^ Division of Epidemiology Department of Health Informatics & Public Health School of Public Health Tohoku University Graduate School of Medicine Sendai Japan

**Keywords:** alcohol drinking, cancer survivor, cross‐sectional study, Japan, prevalence, smoking

## Abstract

**Background:**

We determined the prevalence of current cigarette smokers and alcohol drinkers among cancer survivors and subjects with no history of cancer in Japan and compared the findings with nationally representative studies in other countries.

**Methods:**

We conducted a cross‐sectional study of baseline data from a prospective cohort study. A self‐administered questionnaire was surveyed during 2013–2015 with residents aged ≥20 years attending a community‐based cardiometabolic screening program in Miyagi prefecture in north‐eastern Japan. Subjects with past cancer histories were classified as cancer survivors. Sex‐specific, age‐standardized prevalence of current smokers, and drinkers were calculated. Age‐adjusted prevalence ratios (PRs: the cancer survivors’ rate divided by the rate of subjects with no history of cancer) and 95% confidence intervals (CIs) were estimated with log‐binomial regressions.

**Results:**

36,786 subjects, including 2760 cancer survivors, responded and provided usable information (58.9% of recruited subjects). For men, the age‐standardized prevalence of current smokers and drinkers among survivors was 18.8% and 74.4%, respectively, with an age‐adjusted PR (95%CI) of 0.76 (0.66–0.86, *p *< 0.001) and 0.95 (0.91–0.98, *p *= 0.002), respectively. For women, the figures were 6.1%, 37.9%, 0.84 (0.67–1.06, *p *= 0.138) and 0.96 (0.90–1.03, *p *= 0.313), respectively. The U.S., the U.K, and Australian studies generally showed no substantially lower prevalence of current smokers or drinkers in survivors than in subjects with no history of cancer (PR ≥ 0.75), while Korean studies did (PR < 0.75).

**Conclusions:**

A considerable proportion of Japanese cancer survivors, especially men, remained currently smoking and drinking. Consistent with Western studies, the rates were not substantially lower than those among subjects with no history of cancer.

## Graphical abstract

Japanese cancer survivors were not substantially less likely to be current smokers or alcohol drinkers compared with cancer‐free persons. A systematic review of nationally representative surveys in other countries, except Korea, observed similar findings.

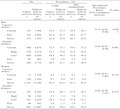

## 1 INTRODUCTION

Health promotion and disease prevention, which addresses issues including cigarette smoking and alcohol drinking, constitute an integral part of comprehensive cancer survivorship care.^1^ Cancer survivors are at higher risk of developing and dying from subsequent primary cancers, compared with the general population.^2^ Smoking^3^ and alcohol^4^, ^5^ are associated with higher risk for many forms of cancers and all‐cause mortality.

In an attempt to quantify the magnitude of these challenges at a national level, the prevalence of current smokers and drinkers among cancer survivors has been estimated and compared with the rates among persons without cancer histories in studies from several countries which analyzed survey data from nationally representative samples.^6^, ^7^, ^8^, ^9^, ^10^, ^11^, ^12^, ^13^, ^14^, ^15^, ^16^, ^17^ In Japan, studies have reported the crude prevalence of smokers and drinkers among cancer survivors and subjects with no history of cancer as part of cross‐sectional^18^ or prospective cohort studies.^19^ However, no study in Japan has focused specifically on a comparison of the age‐adjusted prevalence of current smokers and drinkers between cancer survivors and subjects with no history of cancer, either using a nationally representative sample or a nonrepresentative sample.

We, therefore, conducted this study to determine and compare the prevalence of current smokers and drinkers between cancer survivors and subjects with no history of cancer among participants in a community‐based cardiometabolic screening program in Miyagi prefecture in north‐eastern Japan. We further compared our findings with those of nationally representative surveys in other countries.

## 2 MATERIALS AND METHODS

### 2.1 Study design and subjects

We conducted a cross‐sectional study using data from a baseline questionnaire survey for the Tohoku Medical Megabank Community‐Based Cohort Study, a prospective cohort study of residents in Miyagi and Iwate prefectures, the north‐eastern part of Japan, affected by the Great East Japan Earthquake in 2011. The study protocol was approved by the institutional review board of the Tohoku University Tohoku Medical Megabank Organization. Details of the cohort study design have been described previously.^20^, ^21^ We used data obtained from the Miyagi prefecture for the present analysis.

Briefly, eligible subjects were 62,439 residents in 28 municipalities of Miyagi prefecture, aged 20 years and over as of the fiscal year 2013–2015 (from April 2013 to March 2016), who participated in an annual screening program for cardiometabolic diseases conducted by the municipalities. Municipal governments in Japan are mandated by national legislation to offer annual screening for cardiometabolic diseases for residents aged 40–74 years who are not offered similar programs at their workplace or through other opportunities.^22^ Municipal governments usually provide the examinations for residents aged 20–39 or 75 and over who are asked to participate. The screening examinations are generally conducted at local community centers and include the measurements of weight, height, waist circumference and blood pressure, blood testing (glucose, triglycerides, cholesterols, etc.), and urine testing (glucose, protein, etc.).

As part of the baseline survey for the prospective study, participants in the screening program were invited to respond to a self‐administered questionnaire. Examples of items asked in the questionnaire were demographics and other basic variables (sex, age, education, marital status, self‐rated health, etc.), past histories of diseases (including cancer), health habits (cigarette smoking, alcohol drinking, diet, etc.), and psychologic symptoms (distress, depression, etc.). Of the 62,439 eligible subjects, 37,874 provided informed consent to participate in the study and returned the self‐administered questionnaire.

### 2.2 Definition of cancer survivors

The information on all variables analyzed in this study was collected from the self‐administered questionnaire. The questionnaire asked whether subjects had ever been diagnosed with cancer by physicians and presented a list of 17 cancer sites as well as an open‐ended space for “other” cancer sites. Subjects with a physician diagnosis of 17 cancer sites were asked to check the corresponding boxes, and those with the diagnosis of “other” cancer sites were asked to fill them in manually. “Cancer survivors” in this study were defined as subjects who reported a history of physician diagnosis for one or more cancer sites. The questionnaire did not ask about the details of the cancer history (the date of diagnosis, the process of diagnosis [i.e., via screening or symptom], the extent of disease progression at diagnosis, types of treatments, etc.).

### 2.3 Smoking and drinking

For smoking, subjects were first asked whether they had ever smoked ≥100 cigarettes during their lifetime. Those who responded affirmatively were classified as ever smokers, and those who responded otherwise were classified as never smokers. Ever smokers were further asked whether they currently smoked cigarettes, and those who responded affirmatively were classified as current smokers and those who responded otherwise were classified as past smokers.

For alcohol drinking, subjects were first asked to choose one out of four categories: consume alcohol currently (once or more per month), consumed in the past but quit currently, never or almost never consumed, or unable to consume due to life‐long constitution (i.e., genetic makeup). Subjects who chose the first and second response were classified as current and past drinkers, respectively, and those who chose the third or fourth response were classified as never drinkers. Current drinkers were further asked about the average frequency of consumption for each of six beverages (sake, beer, whisky, wine, etc.) and the amounts of intake per occasion (the numbers of units of sake, bottles of beer, glasses of wine, etc.). The average amount of alcohol consumption per day was calculated based on these responses. Current drinkers who consumed ≥40g per day of alcohol in men and ≥20g per day in women were classified as current drinkers with high consumption, and those who consumed less were classified as current drinkers with low consumption. Cut‐off values for classifying high or low consumption were consistent with those used in the National Health and Nutritional Survey (NHNS) in Japan, an annual survey of diet and health behaviors in a nationally representative sample of the Japanese population aged 20 years and over.^23^

### 2.4 Statistical analyses

Of the 37,874 respondents to the self‐administered questionnaire, we excluded subjects who had missing or incomplete responses on smoking or drinking (*n* = 1076) and those with implausible responses on history of cancers (*n* = 12) (e.g., cervical cancer in male subjects). The remaining 36,786 subjects (14,089 men and 22,697 women) (58.9% of the 62,439 eligible subjects) were used for our analyses, including 2760 cancer survivors (1214 men and 1546 women) and 34,026 subjects with no history of cancer (12,875 men and 21,151 women). All analyses were conducted for men and women separately.

For smoking and drinking variables, crude and age‐standardized prevalence were calculated and compared between cancer survivors and subjects with no history of cancer. Age‐standardization was performed using as reference standard the sex‐specific age distributions of cancer survivors and subjects with no history of cancer combined. Furthermore, age‐adjusted prevalence ratios (PRs), or the prevalence of current smokers or drinkers among cancer survivors divided by the corresponding rate in subjects with no history of cancer, their 95% confidence intervals (CIs), and P‐values were estimated by log‐binomial regression models using the SAS GENMOD Procedure.^24^ The *p*‐values were two‐sided, and statistical significance was determined at the *p* < 0.05 level. All analyses were performed using version 9.4 of SAS software (SAS Institute Inc.).

### 2.5 Comparison with studies in other countries

We used PubMed to search and identify original articles in the English language that reported the prevalence of current smokers or drinkers among cancer survivors and compared the rates with those among subjects with no history of cancer using nationally representative samples. We used two search strings: “(smoking OR cigarette) AND cancer survivors” and “(drinking OR alcohol) AND cancer survivors.” We also looked for citations of the original articles identified through PubMed searches. Characteristics of each study were extracted and summarized, including country, the name of the study, survey years, the number of cancer survivors, and subjects with no history of cancer, age, sex, race (if applicable), as well as the prevalence of current smokers and drinkers among cancer survivors and subjects with no history of cancer. For each study, we calculated the prevalence ratio as the prevalence of current smokers or drinkers among cancer survivors divided by the corresponding rate among subjects with no history of cancer and then compared them across the studies including this study.

## 3 RESULTS

Table 1 shows the distributions of cancer sites among cancer survivors. For men, the four major cancer sites were prostate, stomach, colorectum, and lung. For women, they were breast, cervix, colorectum, and stomach. Some survivors had a history of cancers in two or more sites (8.9% in men and 6.1% in women).

**TABLE 1:** Numbers of cancer survivors and cancer sites

	Men			Women	
	No.	%		No.	%
No. of cancer survivors^a^	1214			1546	
Cancer sites^a^					
Prostate	399	29.6	Breast	601	36.0
Stomach	352	26.1	Cervix	243	14.6
Colorectum	322	23.9	Colorectum	225	13.5
Lung	81	6.0	Stomach	174	10.4
Malignant lymphoma	44	3.3	Lung	100	6.0
Kidney	36	2.7	Uterine body	88	5.3
Liver	25	1.9	Ovarian	76	4.6
Others	90	6.7	Others	161	9.7
Total	1349	100.2		1668	100.1
No. of cancer sites per survivor					
1	1106	91.1		1451	93.9
≥2	108	8.9		95	6.1

^a^ The number of cancer survivors was exceeded the number of cancer sites because some cancer survivors had histories of cancers in two or more sites.

The cancer survivors tended to be older than the subjects with no history of cancer, with the mean (SD) age of 67.9 (4.9) and 61.4 (11.0) in men, respectively, and 62.4 (8.9) and 57.8 (12.0) in women, respectively. Table 2 presents age‐standardized prevalence of current smokers and drinkers among cancer survivors and subjects with no history of cancer, along with age‐adjusted PRs. A considerable proportion of cancer survivors, especially men, were current smokers and drinkers, with the age‐standardized prevalence of 18.8% and 74.4% in men, respectively, and 6.1% and 37.9% in women, respectively. Cancer survivors were not substantially less likely to be current smokers or drinkers compared with subjects with no history of cancer, with the age‐adjusted PR (95% CI) of 0.76 (0.66–0.86, *p* < 0.001) and 0.95 (0.91–0.98, *p* = 0.002) in men, respectively, and 0.84 (0.67–1.06, *p* = 0.138) and 0.96 (0.90–1.03, *p* = 0.313) in women, respectively, although the findings in men were statistically significant.

**TABLE 2:** Prevalence and prevalence ratios for cigarette smokers and alcohol drinkers among cancer survivors and subjects with no history of cancer

	No.		Prevalence (%)				Age‐adjusted Prevalence Ratio^b^ (95% Confidence Interval)	*p*‐value
	Cancer survivors	Subjects with no history of cancer	Crude		Age‐standardized^a^			
			Cancer survivors	Subjects with no history of cancer	Cancer survivors	Subjects with no history of cancer		
Men								
Cigarette smokers								
Current	197	3496	16.2	27.2	18.8	26.7	0.76 (0.66–0.86)	<0.001
Past	745	6086	61.4	47.3	54.5	47.7		
Never	272	3293	22.4	25.6	26.7	25.6		
Alcohol drinkers								
Current	880	9674	72.5	75.1	74.4	75.3	0.95 (0.91–0.98)	0.002
High^c^	276	3214	22.7	25.0	23.5	24.9		
Low^d^	604	6460	49.8	50.2	51.0	50.4		
Past	85	485	7.0	3.8	7.5	3.8		
Never	249	2716	20.5	21.1	18.1	20.9		
Women								
Cigarette smokers								
Current	71	1538	4.6	7.3	6.1	7.1	0.84 (0.67–1.06)	0.138
Past	150	2104	9.7	9.9	12.7	9.8		
Never	1325	17,509	85.7	82.8	81.3	83.0		
Alcohol drinkers								
Current	541	8182	35.0	38.7	37.9	38.5	0.96 (0.90–1.03)	0.313
High^c^	88	1543	5.7	7.3	7.0	7.2		
Low^d^	453	6639	29.3	31.4	31.0	31.3		
Past	40	362	2.6	1.7	2.9	1.7		
Never	965	12,607	62.4	59.6	59.2	59.8		

^a^ Percentages were age‐standardized using as reference standard the sex‐specific, age distributions of cancer survivors and subjects with no history of cancer combined.

^b^ Adjusted for age with log‐binomial regression models.

^c^ Defined as daily alcohol consumption of ≥40 g for men and ≥20 g for women.

^d^ Defined as daily alcohol consumption of <40g for men and <20g for women.

As summarized in Tables 3 and 4, we identified 12 studies from four countries, including the United States,^6^, ^7^, ^8^, ^9^, ^10^, ^11^, ^12^ the United Kingdom,^13^ Australia,^14^ and Korea,^15^, ^16^, ^17^ which analyzed data from nationally representative surveys to estimate the prevalence of current smokers and drinkers among cancer survivors and subjects with no history of cancer. For current smokers (Table 3), studies in Western countries generally found that the prevalence was not substantially lower, or even higher, in cancer survivors than in subjects with no history of cancer (PR ≥ 0.75). In contrast, two of three Korean studies^15^, ^17^ showed that the prevalence was lower in cancer survivors than in subjects with no history of cancer (PR < 0.75). Our results (age‐adjusted PR = 0.76 for men and 0.84 for women) were consistent with those of Western studies.

**TABLE 3:** Prevalence of current smokers among cancer survivors and subjects with no history of cancer in the present study and other studies using nationally representative samples

Country	Study	Year of survey	Age	Race/Age group/Disease status	Sex	No. of subjects		Prevalence (%)			Prevalence ratio^a^	Ref.
						Cancer survivors	Subjects with no history of cancer	Crude/Age‐standardized	Cancer survivors	Subjects with no history of cancer		
Japan	Present study	2013–2016	≥20		M W	1214 1546	12,875 21,151	A	18.8 6.1	26.7 7.1	0.76 0.84	–
0	BRFSS	2013	≥18		M, W	47,139	407,191	C	16.1	18.6	0.87	6
United States	BRFSS (Massachusetts)	2006–2008	≥18		M W	516 1154	6709 11,488	A	24.0 24.0	18.5 15.5	1.30 1.55	7
United States	NHIS	2005–2007	≥18	White AA Other races	M, W	2380 276 106	56,150 11,753 5156	C	18.6 21.4 27.3	20.8 20.9 15.9	0.89 1.02 1.72	8
United States	NHIS	2000	≥18	18–39 40–69 ≥65	M, W	1646	30,700	C	37.7 25.6 7.7	26.2 25.3 10.1	1.44 1.01 0.76	9
United States	NHANES	1999–2008	≥20		M, W	2188	22,441	A	18.3	24.7	0.74	10
United States	NHIS	1998–2001	≥18		M, W	7384	121,347	A	20.2	23.6	0.86	11
United States	NHIS	1998–2000	≥18		M, W	4878	90,737	A	19.7	23.8	0.83	12
United Kingdom	SHS	1995, 1998, 2003 and 2008	≥45		M, W	922	14,103	C	21.0	28.0	0.75	13
Australia	NHS	2001	≥18		M, W	968	5808	C	21.3	18.9	1.13	14
Korea	KNHANES Ⅳ and V	2007–2012	≥20		M, W	1153	36,451	A	9.2	26.5	0.35	15
Korea	KNHANES Ⅳ and V	2007–2012	≥20		M, W	433	30,721	A	18.2	23.0	0.79	16
Korea	KNHANES Ⅳ	2007–2009	≥19	Noncancer chronic disease Noncancer nonchronic disease	M, W	504	5944 10,863	A	9.6 9.6	20.8 30.1	0.46 0.32	17

Abbreviations: A, age‐standardized; AA, African American; BRFSS, Behavioral Risk Factor Surveillance System; C, crude; KNHANES, Korean National Health and Nutrition Examination Survey; M, Men; NHANES,NHIS, National Health Interview Survey. National Health and Nutrition Examination Survey; NHS, National Health Survey; SHS, Scottish Health Surveys; W, Women.

^a^ Prevalence ratio is the prevalence in cancer survivors divided by the prevalence in subjects with no history of cancer.

**TABLE 4:** Prevalence of current drinkers among cancer survivors and subjects with no history of cancer in the present study and other studies using nationally representative samples

Country	Study	Year of survey	Age	Race/age group	Sex	No. of subjects		Prevalence (%)				Prevalence ratio^a^	Ref.
						Cancer survivors	Subjects with no history of cancer	Crude/Age‐standardized		Cancer survivors	Subjects with no history of cancer		
Japan	Present study	2013–2016	≥20		M W	1214 1546	12,875 21,151	A	Current Current	74.4 37.9	75.3 38.5	0.95 0.96	–
United States	BRFSS	2013	≥18		M, W	47,139	407,191	C	Binge^b^ Heavy^b^	8.3 5.1	17.5 6.0	0.47 0.85	6
United States	BRFSS (Massachusetts)	2006–2008	≥18		M W	516 1154	6709 11,488	A	Heavy^c^	8.4 9.3	6.3 5.0	1.33 1.86	7
United States	NHIS	2005–2007	≥18	White AA Other race	M, W	2380 276 106	56,150 11,753 5156	C	Alcohol use^d^	17.9 6.1 17.2	15.7 8.7 7.8	1.14 0.70 2.21	8
United States	NHIS	2000	≥18	18–39 40–69 ≥65	M, W	1646	30,700	C	Risky^e^	15.3 7.8 4.1	13.8 8.8 3.6	1.11 0.89 1.14	9
UnitedStates	NHIS	1998–2001	≥18		M, W	7384	121,347	A	Current^f^ Current heavy/moderate^f^ Current light/ infrequent^f^	52.4 16.3 36.1	62.5 18.9 43.6	0.84 0.86 0.83	11
Australia	NHS	2001	≥18		M, W	968	5808	C	High^g^ Moderate^g^	24.0 34.1	21.4 33.8	1.12 1.01	14
Korea	KNHANES Ⅳ and V	2007–2012	≥20		M, W	1153	36,451	A	Current^h^ Heavy^h^ Low to moderate^h^	49.1 9.0 40.3	74.4 19.5 55.0	0.66 0.46 0.73	15
Korea	KNHANES Ⅳ and V	2007–2012	≥20		M, W	433	30,721	A	Alcohol use^i^	29.1	40.1	0.73	16
Korea	KNHANES Ⅳ	2007–2009	≥19	Noncancer chronic disease Noncancer nonchronic disease	M, W	504	5944 10,863	A	Current^j^ Heavy^j^ Current^j^ Heavy^j^	30.9 6.6 30.9 6.6	47.9 12.3 63.1 14.7	0.65 0.54 0.49 0.45	17

Abbreviations: A, age‐standardized; AA, African American; BRFSS, Behavioral Risk Factor Surveillance System; C, crude; KNHANES, Korean National Health and Nutrition Examination Survey; M, Men; NHIS, National Health Interview Survey; NHS, National Health Survey; W, Women.

^a^ Prevalence ratio is the prevalence in cancer survivors divided by the prevalence in subjects with no history of cancer.

^b^ Binge consumption was defined as ≥5 and ≥4 drinks for one occasion for men and women, respectively. Heavy consumption was defined as >2 and >1 drink/day for men and women, respectively.

^c^ The average intake of ≥60 and 30 drinks in the past month for men and women, respectively.

^d^ Defined as ≥2 and 1 drink(s)/day for men and women, respectively.

^e^ Defined as either ≥12 binge drinking episodes (≥5 alcoholic beverages in a single day) during the previous year, or high weekly consumption (≥15 and ≥10 alcoholic beverages for men and women, respectively).

^f^ Current consumption was defined as the sum of current infrequent consumption (12+ drinks in a lifetime, 12+ drinks in 1 year, and 1–11 drinks in the past year), current light consumption (12+ drinks in a lifetime, 12+ drinks in 1 year, and ≤3 drinks per week in past year), current moderate consumption (12+ drinks in a lifetime, 12+ drinks in 1 year, and, for men, more than 3 drinks per week up to 14 drinks per week or, for women, more than 3 drinks per week up to 7 drinks per week) and current heavy consumption (12+ drinks in a lifetime, 12+ drinks in 1 year, and, for men, more than 14 drinks/week in the past year or, for women, more than 7 drinks/week in the past year).

^g^ High consumption was defined as more than 2 standard drinks per day for men and more than 1 standard drink per day for women. Moderate consumption was defined as up to 2 standard drinks per day for men and up to 1 standard drink per day for women.

^h^ Heavy consumption was defined as ≥60g alcohol (7 drinks) for men and ≥40g (5 drinks) for women, twice or more per week.

^i^ More than 1 glass of alcohol/month.

^j^ Heavy consumption was defined as 7 or more drinks on one occasion for men and 5 or more drinks for women.

For current drinkers (Table 4), studies in Western countries generally found that the prevalence was not substantially lower, or even higher, in cancer survivors than in subjects with no history of cancer. In contrast, all three Korean studies^15^, ^16^, ^17^ showed that the prevalence was lower in cancer survivors than in subjects with no history of cancer. Our results (age‐adjusted PR = 0.95 for men and 0.96 for women) were consistent with those of Western studies.

## 4 DISCUSSION

We found that a considerable proportion of Japanese cancer survivors, especially men, remained current smokers and drinkers and that the age‐adjusted prevalence of current smokers or drinkers among cancer survivors was not substantially lower than the rate among subjects with no history of cancer. To the best of our knowledge, this is the first report from Japan which specifically focused on a comparison of the prevalence of current smokers and drinkers between cancer survivors and subjects with no history of cancer.

Previous studies of nationally representative samples in Western countries have been generally consistent with our findings that the prevalence of current smokers or drinkers was not substantially lower in cancer survivors than in subjects with no history of cancer (Tables 3 and 4). The observations in Korean studies^15^, ^16^, ^17^ were notable exceptions in that the prevalence of current smokers and drinkers was considerably lower in cancer survivors than in subjects with no history of cancer.

One strength of this study involves the definition of current alcohol drinkers. Our definition included both subjects who consumed a relatively higher amount of alcohol (≥40 g/day in men and ≥20 g/day in women) and those who consumed a lower amount (<40 g/day in men and <20 g/day in women). In contrast, many previous studies have reported only the prevalence of “binge,”^6^ “heavy,”^6^, ^7^ or “risky”^9^ drinkers and did not report the prevalence of drinkers with lower consumption. Evidence suggests, however, that low alcohol consumption is associated with a higher risk of breast cancer and some other cancers,^4^ while not associated with a lower risk of all‐cause mortality,^25^ and that alcohol use, regardless of amount, leads to an increase in the total health burdens.^5^ The inclusion in our study of drinkers with lower alcohol consumption into the definition of current drinkers would lead to a better quantification for the magnitude of alcohol risks among cancer survivors than merely reporting the prevalence of drinkers with higher consumption. Future studies can contribute better to the evidence base by reporting the prevalence of current drinkers with lower and higher consumption combined.

Several limitations of our study warrant comment. First, the subjects did not comprise a nationally representative sample. In comparison with the nationally representative National Health and Nutrition Survey,^23^ as shown in Supplementary Table S1, the rates of the sex‐ and age‐specific prevalence of current smokers in our study were similar to the rates in the national sample. Age‐specific prevalence of current drinkers with high consumption tended to be slightly higher among men, but not among women, in our study than in the NHNS, possibly reflecting a higher per capita sales of alcohol beverages in the study area compared with the national average.^26^ Therefore, our findings would provide useful estimates for the prevalence of smoking and drinking at the national level (with the reservation for drinking in men) and meaningful comparisons with the observations of nationally representative surveys in other countries.

Second, because our subjects were participants in a cardiometabolic screening program, the prevalence of survivors of all cancers and specific cancer sites could differ from the rates expected from the general population. We could not identify literatures reporting the prevalence of cancer survivors in representative samples from either Miyagi prefecture or Japan with which our findings could have been directly compared. As an alternative, we compared a relative ranking for the prevalence of six most common cancer sites in this study and the ranking for the incidence of corresponding cancer sites in nationwide data in 2014 (mid‐year for our survey).^27^ As shown in Supplementary Table S2, the ranking of prevalent cancer sites in this study was generally consistent with that of national cancer incidence, except prostate cancer in men (first in this study and fourth in the national incidence) and cervical cancer in women (second in this study and sixth in the national incidence) were overrepresented. As possible reasons for the observations, some municipal governments offer the screening for cardiometabolic diseases and screenings for cancers (including cervix, prostate, stomach, colorectum, and lung) at the same dates and venues (such as community centers), so that subjects in this study would have been more likely to participate in cancer screening programs in addition to the cardiometabolic screening program than the general population. This would have led to overrepresentation for the prevalence of screen‐detected cancers in general and prostate cancer in men and cervical cancer in women in particular (with high survival rates of screen‐detected cancer of the two sites) relative to the prevalence expected from the general population. Likely over‐representation of screen‐detected cancers in this study further suggests that our subjects (both cancer survivors and subjects without history of cancer) had higher awareness regarding the benefit of cancer screenings and the harm of risk‐increasing behaviors including smoking and drinking compared with the general population and that cancer survivors in this study would less likely to have remained current smokers or drinkers after cancer diagnosis compared with cancer survivors in the general population. This differential awareness would have resulted in larger differences in the prevalence of current smokers and drinkers among cancer survivors and subjects without a history of cancer in this study than the expected differences between two groups in the general population. Therefore, PRs expected from the general population would likely be closer to unity than the PRs observed in this study (0.76–0.96), implying that the extent of lower prevalence of current smokers and drinkers among cancer survivors relative to subjects without a history of cancer would be even smaller in the general population.

Third, the definition of cancer survivors was based on self‐reports of history for physician diagnosis of cancers. Studies in the Japanese populations showed moderate to high sensitivity and specificity for self‐reports of history of cancer and other chronic diseases against medical records.^28^, ^29^, ^30^ Thus, a serious misclassification between cancer survivors and subjects with no history of cancer in the study subjects seems unlikely.

Fourth, because no information was available for the date of cancer diagnosis, we were not able to determine temporal sequences between cancer diagnosis and cessation of smoking or drinking. Specifically, some past smokers and drinkers might have quite a long time after their cancer diagnoses. In this case, the actual prevalence of current smoking and drinking among cancer survivors at the time of initial cancer diagnosis could be higher than the estimated prevalence, and actual health risks posed by continued smoking and drinking for cancer survivors could likely be larger.

Our findings showed that many Japanese cancer survivors remained currently smoking and drinking. In addition to the addictive properties of nicotine and alcohol that would prevent them from quitting even if they wanted to, other potential barriers against the cessation of smoking and drinking might include the belief of some clinicians that they are inadequately trained to deliver effective treatment for tobacco use,^31^ a lack of knowledge among clinicians and survivors that alcohol is a risk factor for cancer,^32^ as well as the perception that “moderate” alcohol consumption is beneficial to general health.^33^ High‐quality evidence from randomized trials in clinical settings for the efficacy of smoking cessation interventions among cancer survivors has only been emerging.^34^ Along with observational studies aiming to understand barriers in addressing smoking and alcohol problems for cancer survivors and intervention trials assessing the efficacy of cessation programs, continued efforts through descriptive studies would be a priority to accurately quantify and characterize the magnitude of health risks posed by smoking and drinking to cancer survivors in various countries and populations.

In conclusion, this cross‐sectional study of Japanese men and women showed that a considerable proportion of cancer survivors, especially men, remained currently smokers and drinkers. The study also found that the prevalence of current smokers or drinkers was not substantially lower among cancer survivors than the rate among subjects with no history of cancer. Moreover, these findings were generally consistent with previous studies of nationally representative samples in Western countries. Our findings underscore the importance of further incorporating smoking and alcohol cessation programs as an integral part of comprehensive cancer survivorship care, both in Japan and globally.

## 5 INFORMED CONSENT

Informed consent was obtained from all the individual participants included in the study.

## CONFLICT OF INTEREST

The authors declare no conflict of interest.

## AUTHOR CONTRIBUTIONS

**Yuka Nishimoto:** Study conception and design, statistical analysis, interpretation of data, writing‐original draft, critical revision of the article for important intellectual content, and approval of the final version for submission. **Yoshitaka Tsubono:** Study conception and design, statistical analysis, interpretation of data, writing‐original draft, critical revision of the article for important intellectual content, and approval of the final version for submission. **Mana Kogure:** Data collection, preparation, interpretation of data, critical revision of the article for important intellectual content, and approval of the final version for submission. **Tomohiro Nakamura:** Data collection, preparation, interpretation of data, critical revision of the article for important intellectual content, and approval of the final version for submission. **Fumi Itabashi:** Data collection, preparation, interpretation of data, critical revision of the article for important intellectual content, and approval of the final version for submission. **Naho Tsuchiya:** Data collection, preparation, interpretation of data, critical revision of the article for important intellectual content, and approval of the final version for submission. **Naoki Nakaya:** Data collection, preparation, interpretation of data, critical revision of the article for important intellectual content, and approval of the final version for submission. **Kozo Tanno:** Data collection, preparation, interpretation of data, critical revision of the article for important intellectual content, and approval of the final version for submission. **Junichi Sugawara:** Data collection, preparation, interpretation of data, critical revision of the article for important intellectual content, and approval of the final version for submission. **Shinichi Kuriyama:** Data collection, preparation, interpretation of data, critical revision of the article for important intellectual content, and approval of the final version for submission. **Shigeo Kure:** Data collection, preparation, interpretation of data, critical revision of the article for important intellectual content, and approval of the final version for submission. **Ichiro Tsuji:** Data collection, preparation, interpretation of data, critical revision of the article for important intellectual content, and approval of the final version for submission. **Atsushi Hozawa:** Study conception and design, interpretation of data, critical revision of the article for important intellectual content, and approval of the final version for submission.

## ETHICAL APPROVAL

All procedures performed in studies involving human participants were in accordance with the ethical standards of the institutional and/or national research committee and with the 1964 Helsinki declaration and its later amendments or comparable ethical standards. This article does not contain any studies with animals performed by any of the authors.

## Supporting information

Table S1‐S2
