# Supplementary material for: The prevalence of current smokers and alcohol drinkers among cancer survivors and subjects with no history of cancer among participants in a community‐based cardiometabolic screening program in Miyagi prefecture, Japan: a comparison with nationally representative surveys in other countries
Source: Cancer Med. 2021 Dec 1;10(24):9000–11. doi: 10.1002/cam4.4364 (PMC8683559; doi:10.1002/cam4.4364)
Supplement: Supplementary file 1 — Table S1‐S2 [file CAM4-10-9000-s001.docx]

| **SUPPLEMENTARY TABLE 1.** Sex- and Age-specific Prevalence of Current Smokers and Current Drinkers With High Alcohol Consumption in the Present Study and the National Health and Nutritional Survey (NHNS) of Japan, 2014^23^ | | | | | | | | | | | |
| --- | --- | --- | --- | --- | --- | --- | --- | --- | --- | --- | --- |
|  | Men | | | | |  | Women | | | | |
|  | Present study | |  | NHNS | |  | Present study | |  | NHNS | |
|  | No. of subjects | **%** |  | No. of subjects | **%** |  | No. of subjects | **%** |  | No. of subjects | **%** |
|  |  |  |  |  |  |  |  |  |  |  |  |
| Current smoker | |  |  |  |  |  |  |  |  |  |  |
| 20-29 | 58/195 | 29.7 |  | 95/259 | 36.7 |  | 58/399 | 14.5 |  | 38/324 | 11.7 |
| 30-39 | 312/693 | 45.0 |  | 199/449 | 44.3 |  | 322/2,106 | 15.3 |  | 69/483 | 14.3 |
| 40-49 | 453/1,048 | 43.2 |  | 239/541 | 44.2 |  | 377/2,514 | 15.0 |  | 79/617 | 12.8 |
| 50-59 | 669/1,745 | 38.3 |  | 197/541 | 36.4 |  | 433/4,387 | 9.9 |  | 75/612 | 12.3 |
| 60-69 | 1,697/6,988 | 24.3 |  | 275/845 | 32.5 |  | 356/10,018 | 3.6 |  | 57/902 | 6.3 |
| ≥70 | 504/3,420 | 14.7 |  | 140/925 | 15.1 |  | 63/3,273 | 1.9 |  | 28/1,128 | 2.5 |
| Current drinker with high alcohol consumption^a^ | |  |  |  |  |  |  |  |  |  |  |
| 20-29 | 12/195 | 6.2 |  | 15/130 | 11.5 |  | 24/399 | 6.0 |  | 6/121 | 5.0 |
| 30-39 | 127/693 | 18.3 |  | 42/258 | 16.3 |  | 231/2,106 | 11.0 |  | 19/182 | 10.4 |
| 40-49 | 252/1,048 | 24.0 |  | 73/367 | 19.9 |  | 321/2,514 | 12.8 |  | 27/269 | 10.0 |
| 50-59 | 552/1,745 | 31.6 |  | 92/369 | 24.9 |  | 440/4,387 | 10.0 |  | 15/210 | 7.1 |
| 60-69 | 1,826/6,988 | 26.1 |  | 119/588 | 20.2 |  | 526/10,018 | 5.3 |  | 10/262 | 3.8 |
| ≥70 | 721/3,420 | 21.1 |  | 57/497 | 11.5 |  | 89/3,273 | 2.7 |  | 3/174 | 1.7 |
|  |  |  |  |  |  |  |  |  |  |  |  |
| ^a^ Daily alcohol consumption of ≥40g for men and ≥20g for women. | | | | | | | | | | | |

| **SUPPLEMENTARY TABLE 2.** Ranking of Cancer Sites for Prevalence in the Present Study (2013-2015) and for Incidence in the National Cancer Incidence in Japan (2014)27 | | | | | | | | | | | | |
| --- | --- | --- | --- | --- | --- | --- | --- | --- | --- | --- | --- | --- |
| Men | | | | | |  | Women | | | | | |
| Prevalence in the Present  Study (2013-2015) | | |  | National Incidence (2014) | |  | Prevalence in the Present Study (2013-2015) | | |  | National Incidence (2014) | |
| Cancer  Sites | Rank | Prevalence (%) |  | Rank | Age-adjusted Incidence per 100,000 |  | Cancer  Sites | Rank | Prevalence (%) |  | Rank | Age-adjusted Incidence per 100,000 |
|  |  |  |  |  |  |  |  |  |  |  |  |  |
| Prostate | 1 | 2.83 |  | 4 | 58.7 |  | Breast | 1 | 2.65 |  | 1 | 86.7 |
| Stomach | 2 | 2.50 |  | 1 | 74.3 |  | Cervix | 2 | 1.07 |  | 6 | 14.7 |
| Colorectum | 3 | 2.29 |  | 2 | 68.9 |  | Colorectum | 3 | 0.99 |  | 2 | 41.1 |
| Lung | 4 | 0.57 |  | 3 | 62.4 |  | Stomach | 4 | 0.77 |  | 3 | 26.9 |
| Malignant lymphoma | 5 | 0.31 |  | 9 | 14.6 |  | Lung | 5 | 0.44 |  | 4 | 24.9 |
| Kidney | 6 | 0.26 |  | 7 | 15.8 |  | Uterine body | 6 | 0.39 |  | 5 | 16.0 |
